# Body Mass Index and the Risk of Poor Outcome in Surgically Treated Patients With Good-Grade Aneurysmal Subarachnoid Hemorrhage

**DOI:** 10.1227/neu.0000000000001931

**Published:** 2022-03-24

**Authors:** Ilari Rautalin, Seppo Juvela, R. Loch Macdonald, Miikka Korja

**Affiliations:** * Department of Neurosurgery, University of Helsinki and Helsinki University Hospital, Helsinki, Finland;; ‡ Department of Neurological Surgery, University of California San Francisco, Fresno, California, USA

**Keywords:** Aneurysmal subarachnoid hemorrhage, Body mass index, Glasgow Outcome Scale, Mortality, Obesity paradox, Outcome, Surgery

## Abstract

Heterogeneity among study populations and treatment procedures has led to conflicting results on outcome predictors for patients with aneurysmal subarachnoid hemorrhage (aSAH). One such conflicting predictor is body mass index (BMI).

**OBJECTIVE:**

To clarify whether high BMI values protect patients from poor outcome after aSAH, as previously suggested.

**METHODS:**

We surveyed 6 prospective studies conducted in 14 different countries (93 healthcare units) between 1985 and 2016 and pooled the data on surgically treated patients with good-grade (Glasgow Coma Scale 13-15 on admission) aSAH. We calculated BMI for each patient and created 4 balanced categories based on the BMI quartiles of each cohort. We calculated adjusted odds ratios (ORs) with 95% CIs for the 3-month poor outcome (Glasgow Outcome Scale 1-3) by BMI.

**RESULTS:**

The pooled study cohort included 1692 patients with good-grade aSAH (mean age 51 years; 64% female). At 3 months, 288 (17%) had poor outcomes. The risk for poor outcomes increased with increasing BMI values (OR = 1.15 [1.02-1.31] per each standard deviation increase of BMI). The risk for poor outcome was over 1.6 times higher (OR = 1.66 [1.13-2.43]) in the highest BMI category (range 27.1-69.2) compared with the lowest BMI category (range 14.4-23.8). These associations were found in each of the 6 study cohorts in both men and women, regardless of age.

**CONCLUSION:**

Because higher BMI values seem to associate with poor outcomes in surgically treated patients with good-grade aSAH, it seems unlikely that obesity protects patients with aSAH from poor outcomes.

## Graphical abstract

**Figure:**
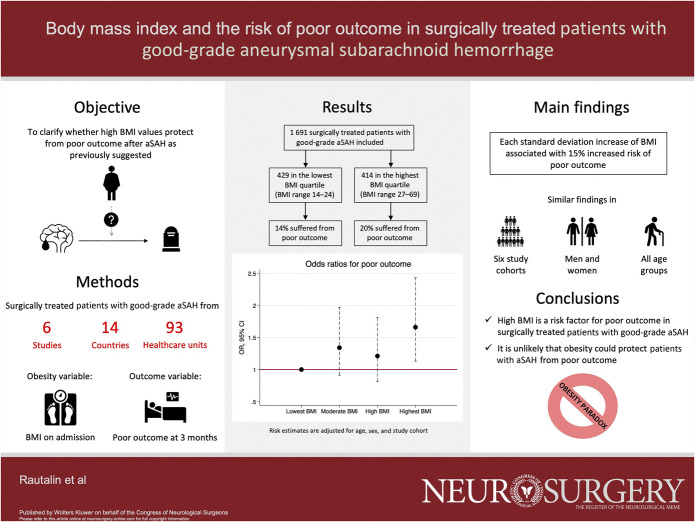

**ABBREVIATIONS:**
IQR: interquartile range
RIA: ruptured intracranial aneurysm.

Numerous studies have examined prognostic factors for outcome after aneurysmal subarachnoid hemorrhage (aSAH).^1^ According to a recent systematic review,^2^ one variable that has been associated with an improved short-term prognosis is a higher body mass index (BMI). The impact of BMI on outcomes after aSAH is of increasing interest because obesity has been related to an improved prognosis (obesity paradox) in several other acute and life-threatening diseases.^3-6^

Previous studies^7-15^ addressing the obesity paradox in aSAH have included hospitalized patients with both poor-grade and good-grade aSAHs. Because half of the patients with the most severe aSAHs, ie, aSAHs that lead to death, die suddenly before hospitalization,^16^ the hospital-based cohorts studying the impact of BMI on the aSAH outcome are inherently biased. Moreover, it is self-evident that patients with poor-grade aSAH are at a high risk for poor outcomes, whereas identifying those patients with good-grade who are at a higher risk for poor outcomes has been tenuous. To clarify the effect of BMI on aSAH outcomes with a more homogenous patient group, we conducted a multicenter study by pooling the data from 6 prospective studies, including hospitalized patients with aSAH from around the world. Pooling enabled us to focus on surgically treated patients with good-grade aSAH on admission—a relatively homogenous subpopulation who could in theory benefit the most from actions that prevent poor outcomes. Based on previous studies,^7,9,10,12,15^ we hypothesized that a higher BMI protects patients with good-grade aSAH from poor outcomes. If true, the escalating global obesity epidemic might affect the overall aSAH outcome figures worldwide.

## METHODS

### Ethical Consideration

Each study received ethical approval from the local institutional authorities, and an informed consent was obtained from all included patients or their relatives. The studies followed the Declaration of Helsinki. Clinical trials (CONSCIOUS-1 [Clazosentan to Overcome Neurological Ischemia and Infarction Occurring after Subarachnoid Hemorrhage Trial], NEWTON-1 [Nimodipine Microparticles to Enhance Recovery While Reducing Toxicity After Subarachnoid Hemorrhage Trial] and IHAST [The Intraoperative Hypothermia for Aneurysm Surgery Trial]) were registered in a public trials registry (ClinicalTrials.gov) before patient enrollment (Refer **Supplemental Digital Content 1**, **Table**, http://links.lww.com/NEU/D19 for further details).

### Study Cohort and Patient Selection

Details of each study have been described before.^17-22^ Summaries of the characteristics and patient selection processes are presented in **Supplemental Digital Content 1**, http://links.lww.com/NEU/D19 and **Supplemental Digital Content 2**, http://links.lww.com/NEU/D20. We pooled the individual patient data from 6 prospective studies (4 clinical trials^18-21^ and 2 cohort studies^17,22^), which included patients with aSAH treated in 93 healthcare centers in 14 countries. The studies included between 73 and 1000 patients, and the data were collected between 1985 and 2016. To avoid selection bias caused by hospital-based settings and strict inclusion criteria of clinical trials, we included only patients with good-grade aSAH on admission. Good-grade was defined as a score on the Glasgow Coma Scale between 13 and 15 (including the World Federation of Neurological Surgeons grades I-III) at the time of hospital admission. Because our primary outcome was measured at 3 months, we also excluded all patients with aSAH who had no outcome information at this follow-up point. Finally, because only 14% of otherwise eligible patients were treated endovascularly (all except 1 of the studies included ≤50 patients who underwent endovascular treatment) and the studies included patients from 4 different decades and dozens of different healthcare centers (endovascular treatment modalities have evolved a lot more than surgical procedures during that period also differing substantially by healthcare units), we decided to focus only on the surgically treated patients with aSAH to further improve the cohort homogeneity.

### Data Collection and Outcome Assessment

We collected information about patients' age, sex, weight, height, smoking habits (nonsmoker/current smoker), and pre-aSAH hypertension status (hypertension diagnosis or antihypertensive medication) (**Supplemental Digital Content 1**, **Table**, http://links.lww.com/NEU/D19). In addition, we extracted data on the aneurysm repair method, location of the ruptured intracranial aneurysm (RIA) and the subarachnoid amount of blood (no/thin, or thick) in the head computed tomography scan. As an outcome assessment, we used the 3-month Glasgow Outcome Score (GOS), which was further dichotomized into favorable (GOS 4-5) and poor (GOS 1-3; severe disability or worse) outcomes. Moreover, we measured the 3-month mortality rate.

### Obesity Assessment

We calculated BMI (kg/m^2^) for each patient. In addition to a continuous assessment (per each standard deviation [SD] increase) of BMI, we also divided the final study cohort into 4 BMI categories based on the analogous BMI quartiles of each individual study cohort: (1) the lowest BMI (the lowest BMI quartile of each study; BMI range 14.4–23.8), (2) moderate BMI (the second BMI quartile of each study; BMI range 21.9–28.3), (3) high BMI (the third BMI quartile of each study; BMI range 24.4–29.4), and (4) the highest BMI (the highest BMI quartile of each study; BMI range 27.1–69.2) (**Supplemental Digital Content 3**, **Table**, http://links.lww.com/NEU/D21 for further details). In addition, we evaluated possible quadratic effects (nonlinear) of BMI on outcome by calculating centered (difference from the median value of BMI) and squared centered BMI values.

### Data Analyses

We used an unconditional logistic regression model to calculate the BMI risk estimates (odds ratios [ORs] and 95% CIs) for poor outcomes and 3-month mortality. In addition to calculating overall estimates for the pooled data set, we also calculated estimates separately for each study and evaluated the cohort heterogeneity by the I^2^-test. In addition to age, sex, and study cohort (a partly adjusted model), we considered factors that associated with both poor outcomes and BMI as possible confounders and included these variables in our fully adjusted multivariable model. To study possible effect modifications caused by sex or age (ie, to study whether these variables alter the association between BMI and aSAH outcomes), we performed stratified subgroup analyses separately for men and women, as well as for younger (≤50 years) and older (>50 years) patients with aSAH. To evaluate the significance of effect modifications caused by age and sex, we calculated *P*-values for multiplicative interactions by using the likelihood ratio test. All statistical analyses were performed by using Stata version 16.1 (Stata Corp).

### Data Availability Statement

Pseudonymized data as well as detailed study protocol and statistical analysis plan can be shared for qualified investigators providing a reasonable request to the corresponding author.

## RESULTS

### Study Cohort

The final study cohort included 1692 surgically treated patients with good-grade aSAH, of which 1675 (99%) had data for the BMI measurement. The mean (median) age of the included patients was 51 years (50), and almost two-thirds (64%) were female. Of the 1692 good-grade patients, 102 (6%) were dead and 288 (17%) had poor outcomes 3 months after aSAH. In the univariable analyses, GOS 1 (deaths) and GOS 1 to 3 (poor outcome) patients were older, were more likely to have a history of hypertension, and had posterior circulation RIAs more often (Table 1). In addition, both GOS 1 and GOS 1 to 3 were associated with the thick aSAH (Table 1).

**TABLE 1.** Patient Characteristics by 3-Month Outcome Status

Characteristic variable	Patient characteristics by 3-mo outcome		ORs (95% CIs) for 3-mo		*P* value for BMI difference by patient characteristics
	Favorable outcome (GOS 4-5)	Poor outcome (GOS 1-3)	Poor outcome (GOS 1-3)	Mortality (GOS 1)	
No. of cases (% by outcome)			—	—	—
Overall	1404 (83.0)	288 (17.0)			
Juvela cohort	152 (82.6)	32 (17.4)			
Enoxaparin trial	103 (78.6)	28 (21.4)			
CONSCIOUS-1	112 (74.2)	39 (25.8)			
NEWTON-1	9 (60.0)	6 (40.0)			
IHAST	856 (85.6)	144 (14.4)			
SHOP	172 (81.5)	39 (18.5)			
Age			—	—	—
Mean (SD)	49.4 (12.4)	56.2 (12.1)			
Median (IQR)	49.0 (41.0-58.0)	56.0 (47.0-66.0)			
Age, n (% by outcome)					.12
<50 y	748 (88.7)	95 (11.3)	(Reference)	(Reference)	
≥50 y	744 (77.3)	228 (22.7)	2.32 (1.77-3.03)	2.17 (1.42-3.33)	
Sex, n (% by outcome)					<.001^a^
Men	514 (83.7)	100 (16.3)	(Reference)	(Reference)	
Women	890 (82.6)	188 (17.4)	1.09 (0.83-1.42)	1.15 (0.75-1.76)	
Hypertension, n (% by outcome)					<.001^a^
No	922 (87.1)	137 (12.9)	(Reference)	(Reference)	
Yes	463 (76.0)	146 (24.0)	2.12 (1.64-2.75)	2.39 (1.59-3.61)	
Missing	19 (79.2)	5 (20.8)			
Smoking, n (% by outcome)					.01^a^
No	637 (82.1)	139 (17.9)	(Reference)	(Reference)	
Yes	765 (84.1)	145 (15.9)	0.87 (0.67-1.12)	0.98 (0.65-1.47)	
Missing	2 (33.3)	4 (66.7)			
BMI			—	—	—
Mean (SD)	26.0 (4.9)	26.7 (5.2)			
Median (IQR)	25.5 (22.7-28.8)	26.0 (23.4-29.3)			
Missing	15 (1.0)	13 (3.9)			
BMI, n (% by outcome)					—
Lowest (BMI 14.4–23.8)	371 (86.5)	58 (13.5)	(Reference)	(Reference)	
Moderate (BMI 21.9–28.3)	347 (82.4)	74 (17.6)	1.36 (0.94-1.98)	1.42 (0.80-2.50)	
High (BMI 24.4–29.4)	347 (84.4)	64 (15.6)	1.18 (0.80-1.73)	0.75 (0.39-1.45)	
Highest (BMI 27.1–69.2)	331 (80.0)	83 (20.1)	1.60 (1.11-2.31)	1.34 (0.75-2.39)	
Missing	8 (47.1)	9 (52.9)			
Aneurysm location, n (% by outcome)					.02^a^
ICA	423 (84.8)	76 (15.2)	(Reference)	(Reference)	
ACA/ACoA	404 (83.0)	83 (17.0)	1.14 (0.81-1.61)	0.99 (0.57–1.70)	
MCA	458 (83.1)	93 (16.9)	1.13 (0.81-1.57)	1.04 (0.62–1.75)	
Posterior	115 (76.2)	36 (23.8)	1.74 (1.11-2.72)	1.86 (0.96-3.57)	
Missing	4 (100.0)	0 (0.0)			
Amount of SAH, n (% by outcome)					.70
None or thin	695 (86.4)	109 (13.6)	(Reference)	(Reference)	
Thick	699 (79.7)	178 (20.3)	1.62 (1.25-2.11)	1.45 (0.96-2.19)	
Missing	10 (90.9)	1 (9.1)			

ACA, anterior cerebral artery; ACoA, anterior communicating artery; aSAH, aneurysmal subarachnoid hemorrhage; BMI, body mass index; CONSCIOUS, Clazosentan to Overcome Neurological Ischemia and Infarction Occurring after Subarachnoid Hemorrhage Trial; GOS, Glasgow Outcome Score; ICA, internal carotid artery; IHAST, The Intraoperative Hypothermia for Aneurysm Surgery Trial; IQR, interquartile range; MCA, middle cerebral artery; NEWTON, Nimodipine Microparticles to Enhance Recovery While Reducing Toxicity After Subarachnoid Hemorrhage Trial; OR, odds ratio; SAH, subarachnoid hemorrhage; SD, standard deviation; SHOP, The Columbia University SAH Outcomes Project.

a Median BMI was significantly higher in men (*P* < .001, Wilcoxon rank-sum test), hypertensive (*P* < .001, Wilcoxon rank-sum test), and nonsmoking patients (*P* = .01, Wilcoxon rank-sum test), as compared with women, normotensive, and smoking patients, respectively. In addition, patients with RIA in posterior circulation had lower median BMI than patients with RIA in other locations (*P* = .02, Kruskal–Wallis test).

### BMI and Poor Outcomes After aSAH

**FIGURE 1.**
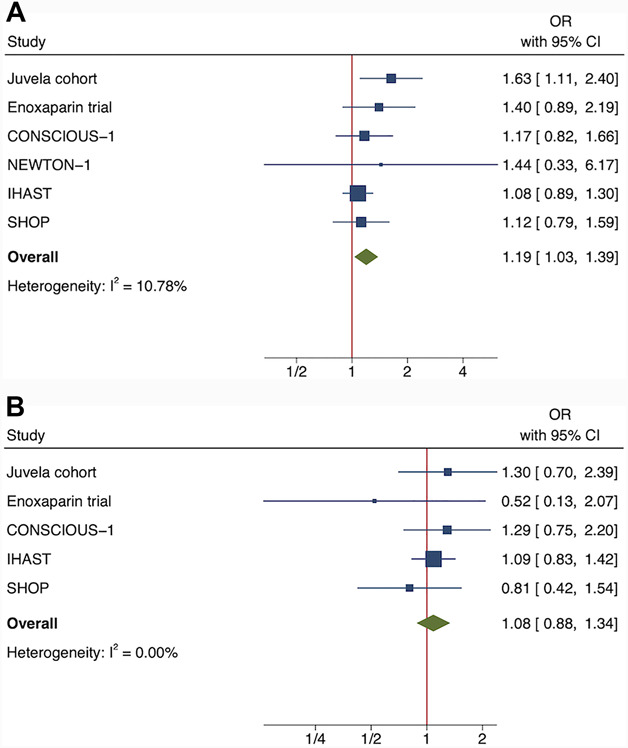
**A** and **B**, A meta-analysis (random effects model) of the association between continuously (per standard deviation) increasing body mass index values and **A**, 3-month poor outcome (GOS 1-3) and **B**, 3-month mortality (GOS 1) after aneurysmal subarachnoid hemorrhage. Risk estimates are adjusted for age. Because there were no mortalities in the NEWTON-1 cohort, we could not include the patients of this cohort (n = 15) into the meta-analysis for mortality. GOS, Glasgow Outcome Score; OR, odds ratio.

**FIGURE 2.**
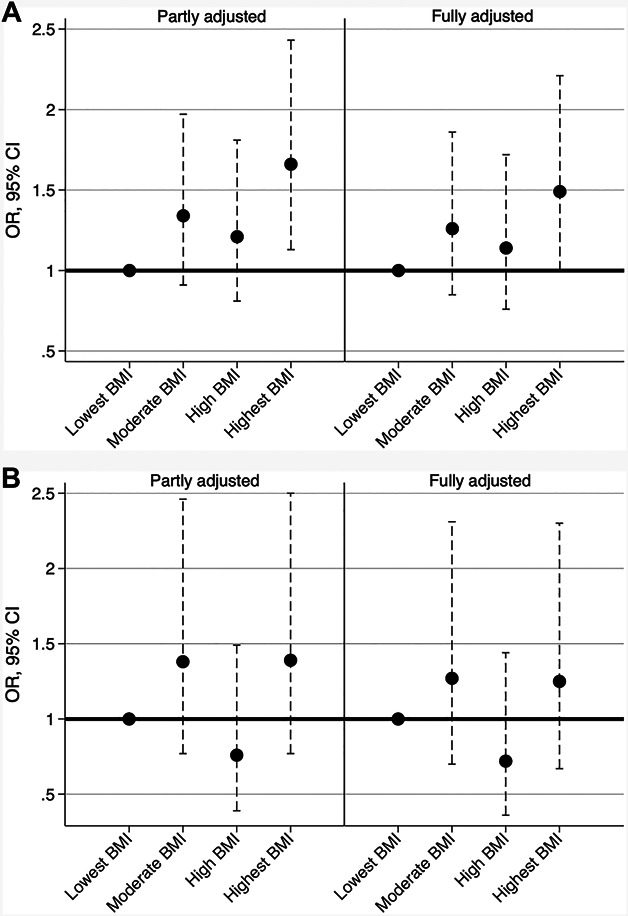
**A** and **B**, Odds ratios with 95% CIs for **A**, 3-month poor outcome and **B**, 3-month mortality by BMI categories. Risk estimates are adjusted for age, sex, and study cohort in the partly adjusted model and for age, sex, study cohort, hypertension, and aneurysm location in the fully adjusted model. We used the low BMI category (lowest BMI quartile of each cohort) as a reference group. BMI, body mass index; OR, odds ratio.

The highest BMI (BMI range 27.1-69.2) associated with poor outcomes (GOS 1-3) in the univariable analysis (Table 1). Both premorbid hypertension and an RIA in the posterior circulation associated with poor outcomes (GOS 1-3) and BMI (hypertension with higher and the posterior RIA location with lower BMI values) (Table 1). Therefore, we considered these 2 factors—in addition to age, sex, and study cohort—as possible confounders in the adjusted multivariable analyses. The regression model, which was adjusted for age, sex, and study cohort, found that each SD (4.7 unit) increase of BMI associated with a 15% (OR = 1.15 [1.02-1.31]) increase in the risk of poor outcomes (GOS 1-3). According to the meta-analysis, the association only differed slightly between the 6 pooled study cohorts (I^2^ = 11% for between-cohort heterogeneity) (Figure 1). After adjusting for age, sex, study cohort, premorbid hypertension, and aneurysm location, the association attenuated slightly (OR = 1.11 [0.98-1.27]). We found no quadratic (nonlinear) effects on this association. In comparison with the lowest BMI category (the lowest BMI quartile of each cohort), the risk for poor outcomes [GOS 1-3] was increased in higher BMI categories, and the risk was most evident in patients with the highest BMI (the highest BMI quartile of each cohort) (Figure 2). The association was found for both men and women, regardless of the age dichotomization (Table 2).

**TABLE 2.** Risk Estimates for 3-Month Poor Outcome and Mortality by BMI Categories in Stratified Subgroup Analyses by Sex and Age Group

Patient subgroup	Poor outcome OR (95% CI)		Mortality OR (95% CI)	
	Partially adjusted^a^	Fully adjusted^b^	Partially adjusted^a^	Fully adjusted^b^
BMI, men				
Lowest	(Reference)	(Reference)	(Reference)	(Reference)
Moderate	1.43 (0.67-3.06)	1.55 (0.70-3.41)	1.74 (0.53-5.74)	2.35 (0.62-8.94)
High	0.90 (0.42-1.93)	0.89 (0.40-1.98)	0.72 (0.20-2.68)	0.80 (0.19-3.42)
Highest	1.65 (0.79-3.43)	1.59 (0.73-3.46)	1.57 (0.48-5.16)	1.89 (0.49-7.21)
BMI, women				
Lowest	(Reference)	(Reference)	(Reference)	(Reference)
Moderate	1.26 (0.80-1.98)	1.10 (0.69-1.76)	1.25 (0.64-2.45)	1.04 (0.52-2.07)
High	1.49 (0.92-2.40)	1.34 (0.82-2.18)	0.81 (0.37-1.81)	0.72 (0.32-1.61)
Highest	1.66 (1.05-2.62)	1.42 (0.89-2.28)	1.36 (0.68-2.72)	1.11 (0.54-2.29)
*P* value for multiplicative interaction (ie, effect modification) by sex	.34	.28	.91	.72
BMI, younger patients (<50 y)				
Lowest	(Reference)	(Reference)	(Reference)	(Reference)
Moderate	1.23 (0.64-2.36)	1.11 (0.57-2.17)	1.44 (0.55-3.76)	1.17 (0.43-3.17)
High	1.40 (0.73-2.69)	1.31 (0.67-2.55)	0.74 (0.23-2.36)	0.63 (0.20-2.03)
Highest	2.01 (1.08-3.76)	1.58 (0.82-3.05)	1.49 (0.57-3.92)	1.04 (0.38-2.85)
BMI, older patients (≥50 y)				
Lowest	(Reference)	(Reference)	(Reference)	(Reference)
Moderate	1.49 (0.93-2.40)	1.42 (0.87-2.31)	1.40 (0.68-2.86)	1.42 (0.68-2.96)
High	1.12 (0.68-1.86)	1.06 (0.64-1.78)	0.75 (0.33-1.72)	0.76 (0.33-1.78)
Highest	1.49 (0.93-2.41)	1.40 (0.86-2.29)	1.27 (0.61-2.65)	1.22 (0.56-2.64)
*P* value for multiplicative interaction (ie, effect modification) by age group	.65	.73	.99	.99

BMI, body mass index; OR, odds ratio.

a Partially adjusted = adjusted for age, sex, and study cohort.

b Fully adjusted = adjusted for age, sex, study cohort, hypertension, and aneurysm location.

### BMI and aSAH Mortality

We found no association between BMI values and 3-month mortality in either partly (OR = 1.06 [0.86-1.30] per each SD increase of BMI) or fully (age, sex, study cohort, premorbid hypertension, and aneurysm location) adjusted models (OR = 1.01 [0.81-1.26] per each SD increase of BMI). The results did not differ between cohorts (I^2^ = 0%) (Figure 1). Risk estimates for 3-month mortality by BMI categories were also inconsistent, with wide CIs (Figure 2). In addition, we found no quadratic effects of BMI on the risk of 3-month mortality. By subgroups of age and sex, the associations of BMI with the 3-month mortality rate remained insignificant (Table 2).

## DISCUSSION

### Key Results

In the pooled analyses of 6 prospective, multinational and multicenter studies—including nearly 1700 surgically treated patients with aSAH—higher BMI values were a significant risk factor for poor outcomes in patients with good-grade aSAH. The association was evident both in men and women—regardless of age—in each of the 6 study cohorts. The risk of poor outcomes for the patients in the highest BMI category attenuated slightly after adjusting the analyses for hypertension, which may relate to the fact that more obese people are more often hypertensive ^23^ and thus at higher risk for poor outcomes. Despite this, the association of an increased risk of poor outcomes in good grade but obese patients with aSAH was relatively strong. In short, our results suggest that the obesity paradox does not exist among surgically treated patients with good-grade aSAH. In fact, the presented results suggest that obesity may be a novel risk factor for poor outcomes in hospitalized and surgically treated patients with good-grade aSAH.

### Interpretation

Previous studies have shown that patients with aSAH with higher BMI values suffer more commonly from various complications, such as postoperative infections,^8^ venous thromboembolisms,^8,24,25^ acute respiratory^7,8^ and renal insufficiencies,^8^ and ischemic lesions.^26^ Given these findings, which are in line with other critical illnesses requiring treatment periods in intensive care units,^27^ our results seem biologically plausible. However, previous studies have also reported that patients with aSAH with higher BMI values have a more favorable clinical outcome^10,12,15^ and lower short-term mortality.^7,9^ These conflicting findings may be attributed, at least partly, to the selection bias, as previously suggested in other stroke types.^28,29^ In aSAH, smoking has been associated with the most severe aSAHs^30^ (sudden deaths) and with lower BMI values in general.^31^ Therefore, hospital-based cohorts may have a selection bias if lean aSAH individuals—who are also more commonly (heavy) smokers—are more frequently excluded from hospital-based cohorts because they die outside of hospitals more often. Moreover, the lean and smoking survivors are more likely to suffer from more severe aSAHs and thus experience more adverse outcomes. In this case, patients with higher BMI values would seem to have a better outcome because of a lower smoking prevalence and less severe aSAH. In our pooled cohort, the smoking prevalence decreased 12% (OR = 0.88 [0.80-0.97]) per each SD increase of BMI, but we were not able to evaluate the association between BMI (or smoking) and aSAH severity because most poor-grade patients were excluded from the study cohorts because of hospital-based study designs and strict inclusion criteria of clinical trials. Among patients with good-grade aSAH, we found no association between smoking and poor outcomes, which relates most likely to survival bias as described previously.^32^ Therefore, according to our original data analysis plan, we did not include smoking to our fully adjusted model as a possible confounder. To avoid some of the shortcomings related to the described selection bias, we focused on patients with good-grade aSAH. Besides selection bias, it is possible that combining endovascularly and surgically treated patients with aSAH has confounded the results of previous studies. To further improve the cohort homogeneity of our study, we focused only on patients with aSAH who underwent surgical aneurysm repair. Finally, the median age of our study cohort was lower in comparison with previous studies reporting obesity paradox in aSAH.^7,9,10,12,15^ Although elderly people may benefit more from a higher BMI than younger adults,^33^ we believe it is unlikely that the slight age difference has a major role in the contradictory findings. In our study cohort, we found no significant difference in the association between BMI and aSAH outcomes by age group. In fact, the results remained similar even for very old (>75 years) patients (data not shown).

### Strengths

To our knowledge, this is the first study evaluating the impact of BMI on the aSAH outcome with prospective data collection at the time of aSAH. Owing to the prospective design of each of the 6 included studies, the data collection may have been more systematic than in previous retrospective studies with data extraction from patient records or hospital-based registries.^7-9,12,15^ In addition, the large sample size enabled us to perform comprehensive analyses for a more homogenous group of good-grade and surgically treated patients with aSAH. Finally, although we were not able to investigate the ethnic differences on the association between BMI and aSAH outcomes (data not available for all study cohort), the pooled studies included patients with aSAH from almost 100 healthcare units around the world, and therefore, our findings may have better external validity than single-center studies.

### Limitations

First, all but one of the included studies^17^ excluded patients with severe comorbidities. Because morbid obesity relates to several comorbidities,^34^ we may have missed some morbidly obese patients. In fact, the largest study (IHAST trial^21^) among the 6 included here excluded all patients with severe obesity (BMI ≥35). However, when we performed sensitivity analyses by excluding the patients of the IHAST trial, the highest BMI category associated even more strongly with poor outcomes (partly adjusted OR = 2.52 [1.42-4.48], as compared with the lowest BMI category). Moreover, to ensure that none of the study cohorts would be underrepresented or overrepresented in a single BMI category, we used a quartile-based approach to classify BMI categories. Nevertheless, the results were very similar even when we used the BMI categories based on the World Health Organization's classification (**Supplemental Digital Content 4**, http://links.lww.com/NEU/D22). Second, because the data about aSAH-related complications (eg, delayed cerebral ischemia, pulmonary embolisms, and kidney dysfunctions) were not collected systematically in every study cohort, we focused on relatively unambiguous outcome variables, namely poor outcomes and mortality. Therefore, future studies with a systematic identification of various complications are needed to clarify the mechanisms for the increased risk of poor outcomes among high BMI patients with aSAH. Third, although excluding endovascularly treated patients with aSAH made the pooled cohort more uniform, future studies are needed to verify whether these findings also apply to endovascularly treated patients. Although the prevalence of endovascular aneurysm repair continues to increase, a large number or even majority of patients with aSAH are still treated surgically, particularly in many non-Western countries such as India,^35^ Pakistan,^36^ Russia,^37^ Indonesia,^38^ and Brazil.^39^ Therefore, our findings may also have a wide impact on current practices, especially considering the global obesity epidemic.

## CONCLUSION

Our results suggest that obesity does not protect surgically treated patients with good-grade aSAH from poor outcomes. In fact, obesity may be a novel risk factor for poor outcomes in this patient group. If these findings also apply to endovascularly treated patients, obese patients with good-grade aSAH may be considered a high-risk patient group for poor outcomes.

## Supplemental Digital Content

**Supplemental Digital Content 1. Table.** Basic characteristics of each 6 studies. **Supplemental Digital Content 2. Figure.** Flow chart of the patient selection process. **Supplemental Digital Content 3. Table.** Detailed characteristics of quartile-based BMI categories. **Supplemental Digital Content 4. Table.** Comparison between the quartile-based and BMI unit–based analyses for the association between BMI and aSAH outcomes (3-month poor outcome and mortality).
